# Expression of Cytosolic Peroxiredoxins in *Plasmodium berghei* Ookinetes Is Regulated by Environmental Factors in the Mosquito Bloodmeal

**DOI:** 10.1371/journal.ppat.1003136

**Published:** 2013-01-31

**Authors:** Benjamin A. Turturice, Michael A. Lamm, James J. Tasch, Angelika Zalewski, Rachel Kooistra, Eric H. Schroeter, Sapna Sharma, Shin-Ichiro Kawazu, Stefan M. Kanzok

**Affiliations:** 1 Department of Biology, Loyola University Chicago, Chicago, Illinois, United States of America; 2 Department of Biology, York University, Toronto, Ontario, Canada; 3 Obihiro University of Agriculture and Veterinarian Medicine, National Research Center for Protozoan Diseases, Obihiro, Hokkaido, Japan; Stanford University, United States of America

## Abstract

The *Plasmodium* ookinete develops over several hours in the bloodmeal of its mosquito vector where it is exposed to exogenous stresses, including cytotoxic reactive oxygen species (ROS). How the parasite adapts to these challenging conditions is not well understood. We have systematically investigated the expression of three cytosolic antioxidant proteins, thioredoxin-1 (Trx-1), peroxiredoxin-1 (TPx-1), and 1-Cys peroxiredoxin (1-Cys Prx), in developing ookinetes of the rodent parasite *Plasmodium berghei* under various growth conditions. Transcriptional profiling showed that *tpx-1* and *1-cys prx* but not *trx-1* are more strongly upregulated in ookinetes developing in the mosquito bloodmeal when compared to ookinetes growing under culture conditions. Confocal immunofluorescence imaging revealed comparable expression patterns on the corresponding proteins. 1-Cys Prx in particular exhibited strong expression in mosquito-derived ookinetes but was not detectable in cultured ookinetes. Furthermore, ookinetes growing in culture upregulated *tpx-1* and *1-cys prx* when challenged with exogenous ROS in a dose-dependent fashion. This suggests that environmental factors in the mosquito bloodmeal induce upregulation of cytosolic antioxidant proteins in *Plasmodium* ookinetes. We found that in a parasite line lacking TPx-1 (TPx-1KO), expression of 1-Cys Prx occurred significantly earlier in mosquito-derived TPx-1KO ookinetes when compared to wild type (WT) ookinetes. The protein was also readily detectable in cultured TPx-1KO ookinetes, indicating that 1-Cys Prx at least in part compensates for the loss of TPx-1 *in vivo*. We hypothesize that this dynamic expression of the cytosolic peroxiredoxins reflects the capacity of the developing *Plasmodium* ookinete to rapidly adapt to the changing conditions in the mosquito bloodmeal. This would significantly increase its chances of survival, maturation and subsequent escape. Our results also emphasize that environmental conditions must be taken into account when investigating *Plasmodium*-mosquito interactions.

## Author Summary

The malaria parasite *Plasmodium* is transmitted by *Anopheles* mosquitoes. Within the midgut of the insect, it is exposed to multiple environmental stresses, including cytotoxic reactive oxygen species (ROS). To avoid destruction, the parasite develops into a motile ookinete capable of leaving the midgut. Yet, ookinete development lasts over several hours and requires the parasite to adapt to an increasingly challenging environment. Here we show that ookinetes of the rodent parasite *Plasmodium berghei* during development in the mosquito midgut increase the expression of the protective antioxidant proteins peroxiredoxin-1 (TPx-1) and 1-Cys peroxiredoxin (1-Cys Prx). This upregulation was also inducible in cultured ookinetes by challenging them with ROS. This suggests that ookinetes actively modulate the expression of their antioxidant proteins in response to the changing conditions in the mosquito. We also found that ookinetes lacking TPx-1 (TPx-1KO) upregulated 1-Cys Prx expression significantly earlier than wild type ookinetes. This indicates that the TPx-1KO parasites compensate for the loss of TPx-1 by altering the expression pattern of the functionally related 1-Cys Prx. The observed dynamic regulation of the cytosolic antioxidant proteins may help the *Plasmodium* ookinete to adapt to rapidly changing environmental conditions and thus to increase the probability of survival, maturation and escape from the mosquito midgut.

## Introduction

Malaria remains a major global threat to human health. One of the reasons for this is the effective transmission and dissemination of the disease-causing protozoan parasite *Plasmodium* by the female *Anopheles* mosquito. For *Plasmodium*, the passage through the mosquito serves two imperative purposes: a) the insect provides the conditions for sexual reproduction and b) it facilitates transport of the new generation of parasites to the next vertebrate host. Following ingestion by the mosquito as part of a blood meal, male and female gametes emerge from their respective host red blood cells (RBCs) to form zygotes. The zygotes convert into highly polarized motile ookinetes, which actively move out of the bloodmeal by invading and crossing the epithelial cell layer to the basal lamina. Here the ookinetes transform into sessile sporozoite-generating oocysts. The sporozoites are released into the hemocoel and eventually invade the salivary glands.

Although the mosquito bloodmeal environment provides the obligatory conditions for parasite gametogenesis and subsequent fertilization [1], it also represents an increasingly challenging environment, which causes the majority of the parasites to perish within the first 24 hours in the mosquito bloodmeal [2], [3]. The reasons for this attrition include mosquito-derived digesting enzymes and the cytotoxic byproducts of their activity, as well as active immune components of the previous vertebrate host [4]. The mosquito also mounts its own innate immune response against the intruding parasite in the form of antimicrobial peptides as well as cytotoxic reactive oxygen (ROS) and reactive nitrogen species (RNS) [5]–[8]. Moreover, when the gametes egress from their host RBCs *Plasmodium* effectively switches its life style from intra- to extracellular parasite and consequently exposes itself directly to the hostile conditions in the mosquito bloodmeal. While gametogenesis and zygote formation occur within an hour of ingestion, the zygote-to-ookinete transformation requires up to 20 hours in the increasingly hostile conditions of the bloodmeal before the mature ookinete is capable of moving through the blood bolus [9].

Yet, despite these adverse conditions, more than 200 million annual clinical episodes of malaria are reported worldwide, the majority of which are direct consequences of mosquito bites [10]. Evidently, a sufficient number of ookinetes survive and escape the mosquito bloodmeal and facilitate malaria transmission. This suggests that ookinetes possess protective mechanisms that help increase the likelihood of their survival. These mechanisms would represent potential targets for transmission intervention strategies.

**Figure 1:**
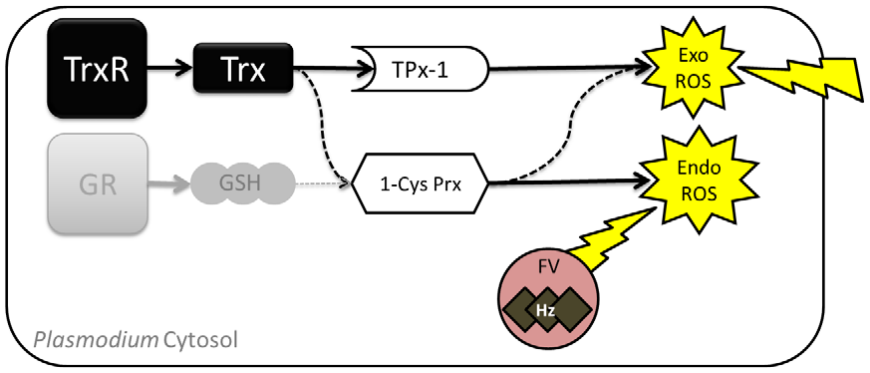
Cytosolic antioxidant systems of *Plasmodium*. Shown is a simplified representation of the Trx- and GSH systems highlighting the roles of the cytosolic peroxiredoxins in asexual *Plasmodium*. The thioredoxin system (black) and the glutathione system (gray) are indicated. Arrows mark the directions of electron flow. Dashed lines indicate that redox reactions have been shown to take place between proteins *in vitro* and are also hypothesized to occur *in vivo*. In asexual parasites the food vacuole (FV) represents a major source for endogenous ROS due to hemoglobin digestion. FV = food vacuole; Exo = exogenous; Endo = Endogenous; Hz = hemozoin.

Cells are generally exposed to ROS and RNS from various sources and therefore possess protective mechanisms involving enzymatic and non-enzymatic antioxidant molecules. In *Plasmodium* two major interdependent antioxidant systems have been described: the thioredoxin system and the glutathione system (Fig. 1) [11], [12]. The glutathione system consists of the small ubiquitous redox active tripeptide glutathione (GSH) and its corresponding reducing enzyme glutathione reductase (GR). The small 12 kDa protein thioredoxin (Trx-1) is central to the thioredoxin system and mainly functions as an electron donor to diverse target proteins *in vitro* and *in vivo*, including key antioxidant enzymes, such as the peroxiredoxins (Prx) (Fig. 1) [13]–[15]. Trx-1 is maintained in its reduced, active state by the flavoenzyme thioredoxin reductase (TrxR) [16].

Peroxiredoxins (Prx) are ubiquitous antioxidant enzymes, which act primarily against cytotoxic peroxide and nitric oxide (NO) compounds [17]. Enzymatic activity depends on the number and position of redox active cysteines that partake in the catalytic thiol-dependent redox mechanism. Peroxiredoxins are hypothesized to play central roles in *Plasmodium* as the parasite lacks the major antioxidant enzymes catalase and glutathione peroxidase [11], [18]. Six members of the Prx family of proteins have been identified and characterized in the *Plasmodium* genomes to date [15]: Two typical thioredoxin-dependent 2-Cys Prx (TPx-1 and TPx-2) [19]–[21], one 1-Cys Prx [22], one atypical glutaredoxin-dependent 2-Cys Prx (AOP) [23], a glutathione peroxidase-like thioredoxin peroxidase (TPx_Gl_) [24] and a novel nuclear Prx (nPrx) [25]. Studies on *P. falciparum* asexual stages have shown that all six genes are expressed. However, the corresponding proteins are localized in different cellular compartments including cytosol (TPx-1, 1-Cys Prx, TPx_Gl_), mitochondria (TPx-2, TPx_Gl_), apicoplast (AOP, TPxGl) and nucleus (nPrx) [25]–[27]. Recent studies in *P. falciparum* have identified TPx-1 and 1-Cys Prx as potential *in vivo* binding partners of Trx-1, leading to the proposal that the biochemical redox pathway [TrxR→Trx-1→TPx-1/1-Cys Prx] may form an important component of the cytosolic antioxidant defense of *Plasmodium* (Fig. 1) [14], [28]. *Plasmodium* TPx-1 is a 2-Cys peroxidase and is hypothesized to be an antioxidant household gene protecting the parasite against exogenous ROS [29]. Disruption of the tpx-1 gene in *Plasmodium* results in an increased sensitivity towards ROS [30]. Although *P. berghei* TPx-1KO parasites exhibit reduced numbers of gametocytes and oocysts, the formation of sporozoites and their infectivity to the liver parenchyma in mice is not affected [31], [32]. 1-Cys Prx protein is highly expressed in asexual *P. falciparum* parasites, specifically in the trophozoite stage [22], [29]. In addition to its antioxidant activity [19], [22] it was also reported that 1-Cys Prx associates with heme, a byproduct of hemoglobin digestion in the food vacuole of the parasite [33]. It was suggested that the cellular function of 1-Cys Prx lies in scavenging endogenous oxidative compounds originating from Hb-digestion during intraerythrocytic development.

Here we report on the transcription- and protein-expression profiles of Trx-1 and the cytosolic thioredoxin-dependent peroxiredoxins TPx-1 and 1-Cys Prx in developing ookinetes of the rodent malaria parasite *Plasmodium berghei*. We compared expression over time between parasites developing in the mosquito bloodmeal to parasites growing under ookinete culture conditions. Our results show that *P. berghei* strongly increases expression of the cytosolic peroxiredoxins both at the transcriptional as well as at the protein level when developing in the mosquito bloodmeal. Peroxiredoxin upregulation was also inducible in ookinete cultures that were previously challenged with exogenous ROS. We furthermore show evidence that 1-Cys Prx expression is altered in TPx-1KO ookinetes possibly to compensate for the loss of TPx-1. We hypothesize that a flexible antioxidant stress response, regulated by factors in the mosquito bloodmeal, contributes to the adaptation of the developing ookinetes to the challenging environment and increases the probability of parasite survival and transmission.

## Results

We hypothesize that the *Plasmodium* ookinete regulates antioxidant protein expression in response to environmental changes in the mosquito bloodmeal in order to maximize the probability of survival, maturation and escape.

### *tpx-1* and *1-cys prx* transcription is upregulated in the mosquito blood meal

**Figure 2:**
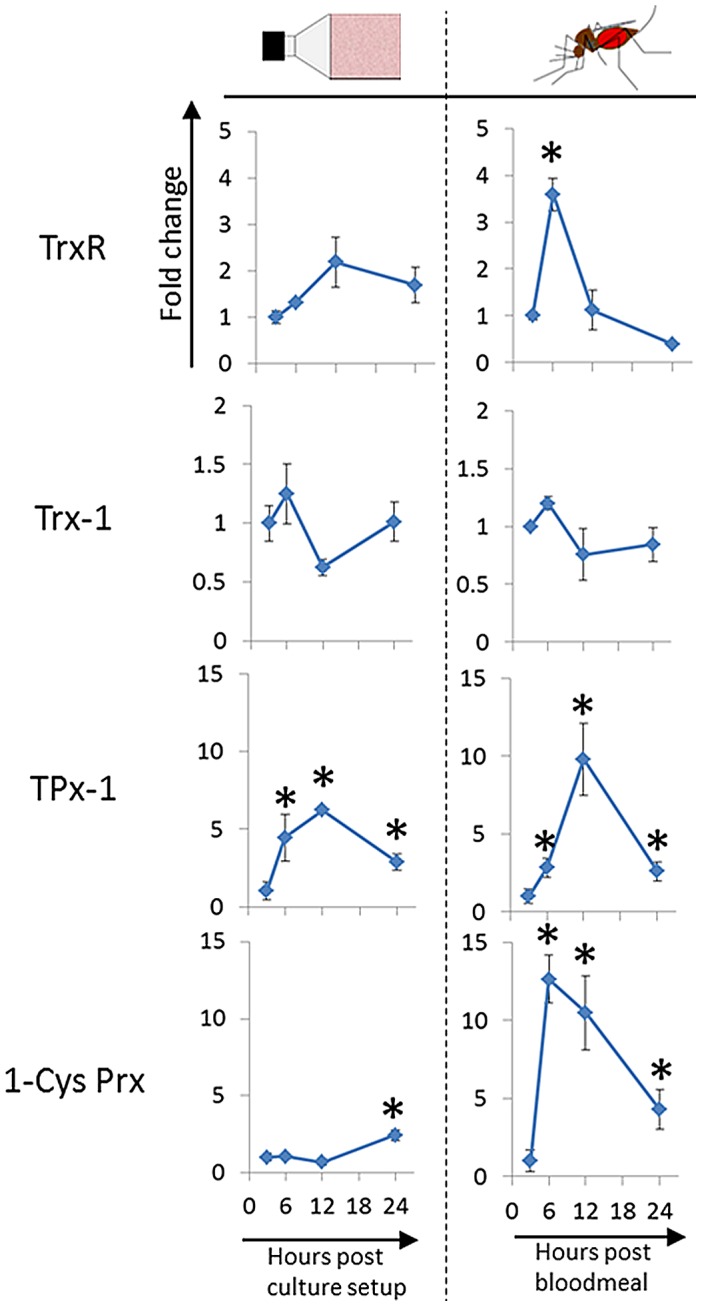
Time dependent transcription profiles of the cytosolic thioredoxin system in culture-derived and mosquito-derived parasites. RT-qPCR data showing relative target gene expression as fold increase over time. The 3-hour time point was used as a reference. All data was normalized against the expression of 18 s rRNA A-type [29], [34]. The Mann-Whitney U test was conducted on each candidate gene from both mosquito-derived and from culture-derived parasites. Significance was assessed at p<0.1(*) due to the small sample sizes. Shown are mean values of 4 independent blood feeding experiments (n = 55 mosquitoes/time point/experiment) and 3 independent ookinete culture setups, respectively. Error bars show STDEV.

To assess the effect of the mosquito bloodmeal on antioxidant enzyme expression in *Plasmodium*, we compared the transcription profiles of *trxr*, *trx-1*, *tpx-1* and *1-cys prx* (Table 1) in parasites that developed either in bloodfed mosquitoes or in ookinete cultures over the course of 24 hours (Figure 2). Female *Anopheles stephensi* mosquitoes were allowed to feed on mice previously infected with *P. berghei* ANKA 2.34. Bloodfed mosquitoes were collected at 3, 6, 12 and 24 hours post infectious bloodmeal (pIBM) and midguts were dissected for total RNA isolation. In parallel, ookinete cultures were prepared and samples were taken at 3, 6, 12 and 24 hours post culture setup for total RNA isolation. Candidate gene expression relative to 18 s rRNA was assessed using a SYBR green based RT-qPCR [34], [35]. Transcription profiles were generated using the 3 hours sample as a reference point. For simplicity, samples from parasites grown in culture will be referred to as *culture-derived* and samples from parasites isolated from dissected bloodfed mosquito midguts as *mosquito-derived*.

**Table 1:** Candidate genes of the cytosolic thioredoxin system of *P. berghei*.

Putative		PlasmoDB		
Function	Abbr.	Gene ID	Uniprot ID	Citations
Thioredoxin reductase	TrxR	PBANKA_082470	Q4Z3E0	[16], [57]
Thioredoxin-1	Trx-1	PBANKA_132090	Q4Z5I8	[16]
Peroxiredoxin-1	TPx-1	PBANKA_130280	Q4Z2P4	[19], [22], [23]
1-Cys peroxiredoxin	1-Cys Prx	PBANKA_122800	Q4YVS0	[23], [35]

It was observed in the transcriptional profiling of culture-derived parasites that only *tpx-1* and *1-cys prx* were significantly modulated over time (Figure 2). *tpx-1* showed significant upregulation at 6 hours (4-fold) and at 12 hours (6-fold) post culture setup. A downregulation was seen between the 12 and 24-hour time points for *tpx-1*. *1-cys prx* transcription was not altered during the first 12 hours but showed an upregulation of 2.5 fold between the 12 and 24 hour timepoints. Unlike the peroxiredoxin genes, *trxr* and *trx-1* transcript abundance did not significantly change under culture conditions. In contrast, mosquito-derived parasites exhibited a greater upregulation of the peroxiredoxin transcripts. *tpx-1* transcripts increased between 3 and 6 hours pIBM (2.5-fold) and more rapidly between 6 and 12 hours (>10-fold) pIBM. This was followed by a steady downregulation between 12 and 24 pIBM. The resulting profile was similar to that observed in culture-derived ookinetes. Conversely, *1-cys prx* expression showed a large increase (>13-fold) between 3 and 6 hours pIBM. Significantly increased transcript levels were also detected at 12 (11-fold) and 24 hours (4-fold) pIBM. *trxr* was initially upregulated, showing a single peak at 6 hours pIBM (3.7-fold). This was followed by an equal downregulation between 6 and 12 hours. *trxr* expression levels were not significantly modified for the rest of the developmental time frame. Comparable to its transcription profile in culture-derived parasites, *trx-1* did not exhibit any significant modulation under bloodmeal conditions. The transcriptional profiles of *1-cys prx*, *tpx-1*, and *trxr* in parasites developing in mosquitoes demonstrate that factors in the bloodmeal environment affect regulation of antioxidant gene expression.

### 1-Cys Prx protein is expressed in mosquito- derived but not in culture- derived ookinetes

The parasite populations investigated in ookinete cultures and bloodfed mosquitoes consisted of mixed developmental stages, including ookinetes. Since it is essential for the ookinete to withstand the increasingly hostile conditions in the bloodmeal, we examined whether the observed upregulation of the peroxiredoxin genes would be directly translated to the functional protein level in developing ookinetes. Samples were taken at 3, 12 and 24 hours from either ookinete cultures or bloodfed mosquitoes. Following fixation, the samples were probed with protein-specific antibodies against Trx-1, TPx-1 or 1-Cys Prx and analyzed using laser scanning confocal or epifluorescence microscopy.

**Figure 3:**
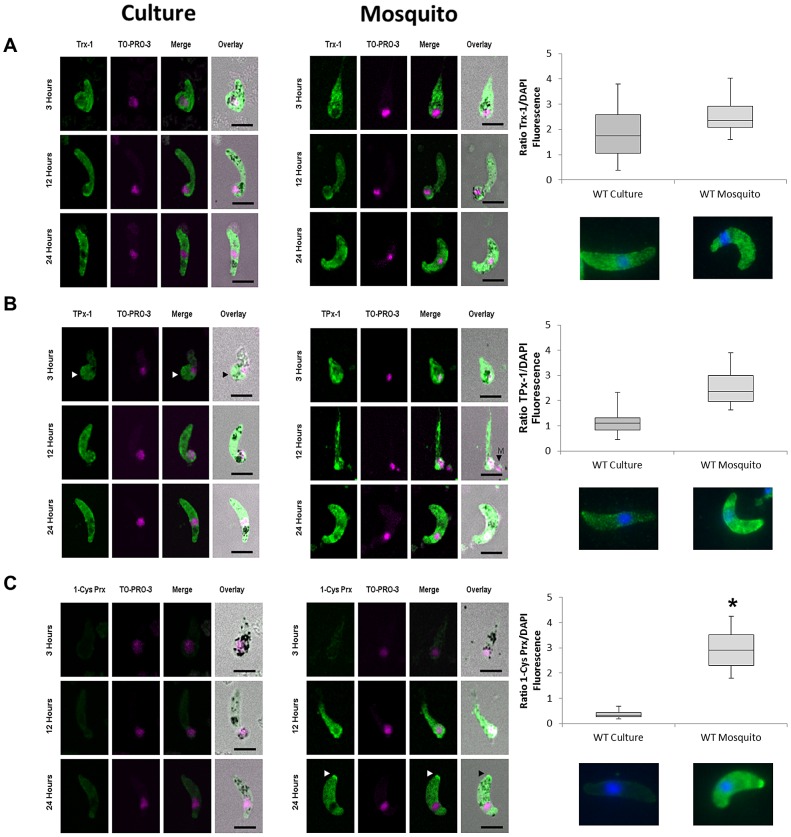
Expression of the Trx-1, TPx-1 and 1-Cys Prx in developing ookinetes from culture or from mosquito bloodmeal. Samples were isolated from ookinete cultures and blood fed mosquitoes at indicated time points and prepared as described in material and methods. Fixed samples were probed with **A**) anti-PbTrx-1, **B**) anti-Pf TPx-1 or **C**) anti-PbTPx-1. Primary antibodies were probed with secondary antibody coupled to AF 488 (Molecular Probes). Samples were counterstained with the nuclear dye TO-PRO-3 (Life technologies). The source of the ookinetes from either ookinete cultures or bloodfed mosquito midguts is indicated. Images are merged and overlaid onto the respective DIC image. White arrows indicate accumulation of Trx-1, TPx-1 or 1-Cys Prx at the apical ends of developing ookinetes. Scale bar = 5 µM. Quantitative *Relative Fluorescence Analysis*: Shown is the median boxed by the first and third quartiles with minimum and maximum values displayed as whiskers. Mann-Whitney U-Test was performed with samples of culture-derived ookinetes compared to mosquito-derived ookinetes (p = 0.4551 for Trx-1; p = 0.1061 for TPx-1; p = 0.0413 for 1-Cys Prx). Below each graph are representative images from IFA-epifluorescence experiments.

Trx-1 was continuously and ubiquitously expressed in all developmental stages of culture-derived as well as mosquito-derived ookinetes (Figure 3A). The uniform distribution of the protein in ookinetes indicated cytosolic localization. Quantitative analysis of relative fluorescence (RF) in mature ookinetes (24 hours) confirmed that there was no significant differential expression of Trx-1 protein between culture-derived and mosquito-derived ookinetes (Figure 3A; median RF = 1.7 vs. 2.1).

TPx-1 was also detected in all ookinete stages in culture-derived as well as mosquito-derived parasites (Figure 3B). The protein was uniformly distributed in the cytosol of the developing ookinetes with the exception of early retort stages from ookinete cultures (3 hour pIBM). Here TPx-1 was detected in the main body of the parasite but not in the apical protrusion. Subsequent developmental stages showed the protein throughout the ookinete. RF analysis showed higher fluorescence intensities in mosquito-derived ookinetes when compared to culture-derived ookinetes (median RF = 1.2 vs. 2.2). However, quantitative analysis revealed that this observed difference was not statistically significant (p = 0.1).

Unlike Trx-1 and TPx-1, 1-Cys Prx was not expressed in early culture-derived ookinetes (3 and 12 hours) and only at low levels in mature ookinetes (24 hours) (Figure 3C). In contrast, in mosquito-derived ookinetes high cytosolic expression of 1-Cys Prx was observed at 12 and 24 hours pIBM (Figure 3C). No signal was detected in early retort stages (3 hours). RF analysis confirmed that the observed higher expression of 1-Cys Prx in mosquito-derived ookinetes when compared to culture-derived ookinetes at 24 hours pIBM was statistically significant (median RF = 0.2 vs. 2.7; p<0.05). Interestingly, an accumulation of 1-Cys Prx protein at the apical ends of mature mosquito-derived ookinetes was observed (Figure 3C). The reason for this localized buildup or its biological significance is not yet clear.

### Upregulation of peroxiredoxins is inducible in ookinete culture

**Figure 4:**
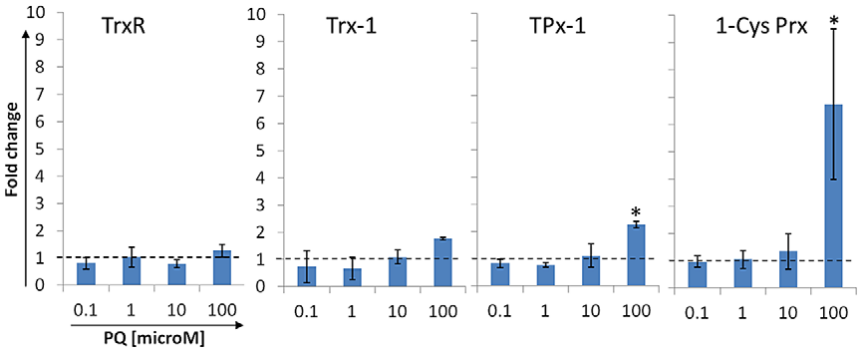
Challenge of ookinete cultures with increasing concentrations of the ROS producing agent paraquat (PQ). RT-qPCR data showing dose-response modulation of target gene expression in ookinete cultures in the presence of increasing concentrations of the superoxide-producing compound Paraquat (PQ, Viologen, Aldrich). Increasing concentrations of PQ were added to 12-hours *P. berghei* ookinete cultures as indicated. Shown are the ratios of normalized relative transcript quantities (RQ) of treated vs. non-treated samples (non-treated RQ = 1, represented by the dashed line). All data were normalized against the expression of 18 s rRNA A-type [29], [34]. (*) indicates statistical significance (p<0.1). Shown are mean values of 3 independent experiments. Error bars indicate STDEV.

Our data indicate that *P. berghei* ookinetes actively respond to changes in the bloodmeal of the mosquito midgut by increasing expression of the cytosolic peroxiredoxins. However, the mosquito midgut represents a complex environment that changes throughout the course of ookinete development. This includes an increase of reactive oxygen species (ROS), such as superoxide radicals [36], [37] and peroxide compounds [6], [7]. We hypothesized that the observed upregulation of cytosolic peroxiredoxins was induced in response to the elevated levels of ROS in the blood bolus. We therefore examined the effect of ROS on peroxiredoxin transcription in ookinete cultures by exposing the parasites to increasing concentrations of paraquat (PQ), a superoxide producer [38], [39]. 12-hours ookinete cultures were challenged with PQ concentrations between 0.1 and 100 µM [30] Parasites were harvested after 45 minutes and total RNA was extracted for RT-qPCR analysis (Figure 4). The ratios of *trxr*, *trx-1*, *tpx-1*, and *1-cys prx* transcription levels in treated and untreated samples were determined. Low and moderate PQ concentrations (0.1 to 10 µM) did not alter transcription levels of any of the candidate genes. At 100 µM PQ the transcription levels of *tpx-1* and *1-cys prx* significantly increased by 2.3-fold and 6.7-fold, respectively. Conversely, *trxr* and *trx-1* did not exhibit significant differential expression in treated and non-treated samples. This data supports our hypothesis that ROS present in the bloodmeal of the mosquito contribute to upregulation of the cytosolic peroxiredoxins in the parasite.

### The expression of 1-Cys Prx protein is altered in TPx-1KO ookinetes

TPx-1 has been hypothesized to play a central role in the antioxidant defense of *Plasmodium* against exogenous stresses [19], [30]. *P. berghei* TPx-1 knock-out parasites (TPx-1KO) exhibit a reduced number of gametocytes and oocysts, while the number of ookinetes and infective sporozoites remain comparable to the wild-type (WT) parasite. Compensatory mechanisms have been suggested [31], [32]. Considering our data we hypothesized that 1-Cys Prx compensates for the loss of TPx-1. We therefore investigated the protein expression profiles of Trx-1 and 1-Cys Prx in culture-derived as well as mosquito-derived TPx-1KO ookinetes.

**Figure 5:**
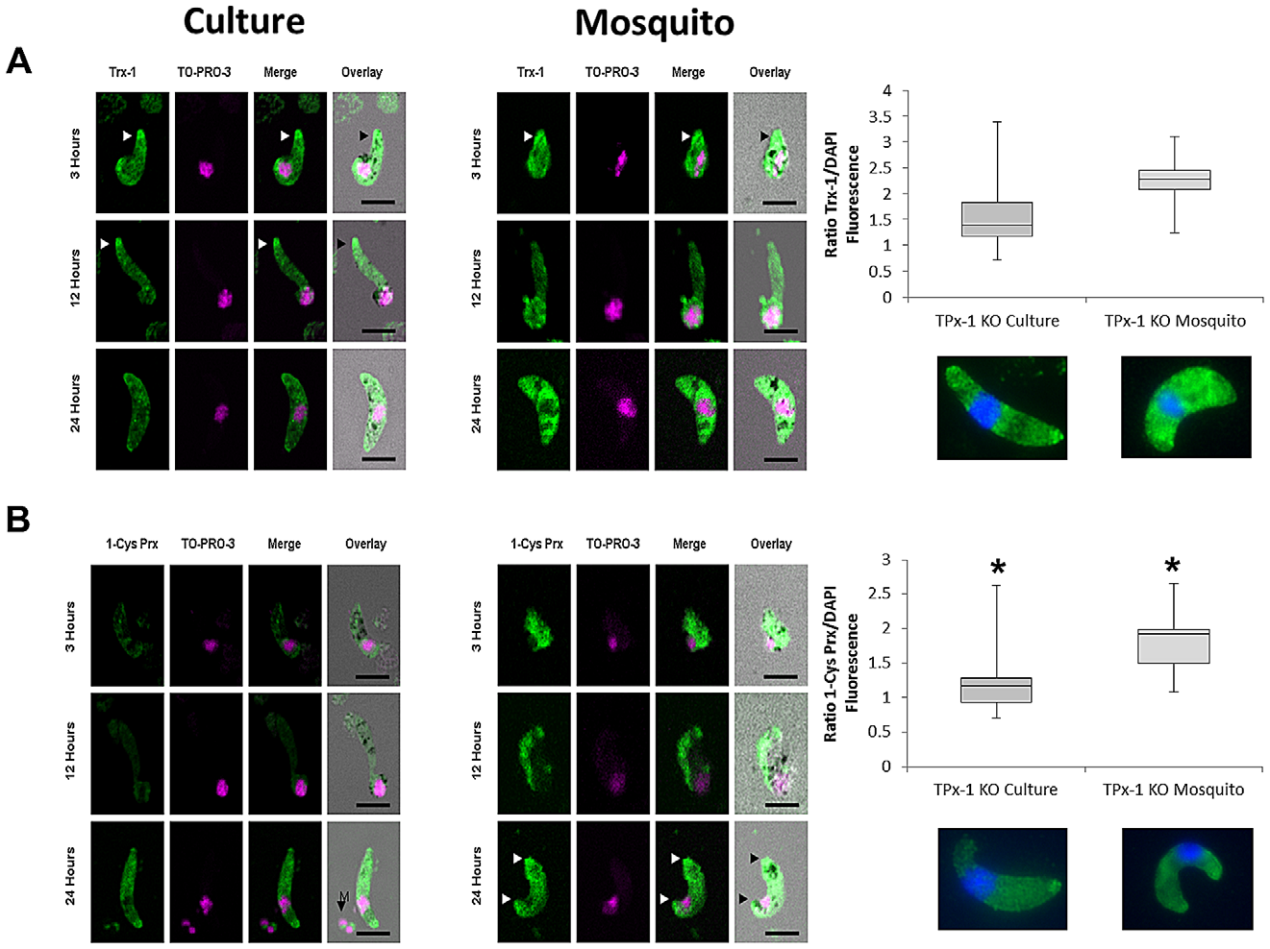
Expression of Trx-1 and TPx-1 in culture-derived and mosquito-derived TPx-1KO ookinetes. TPx-1KO samples were isolated from ookinete cultures and blood fed mosquitoes at indicated time points and prepared as described in material and methods. Fixed samples were probed with **A**) anti-PbTrx-1, **B**) anti-PbTPx-1. Primary antibodies were probed with secondary antibody coupled to AF 488. Samples were counterstained with the nuclear dye TO-PRO-3 (Life technologies). The source of the ookinetes from either ookinete cultures or bloodfed mosquito midguts is indicated. Images are merged and overlaid onto the respective DIC image. White arrows indicate accumulation of Trx-1 or 1-Cys Prx at the apical ends of developing ookinetes. Scale bar = 5 µM. Quantitative *Relative Fluorescence Analysis*: Shown is the median boxed by the first and third quartiles with minimum and maximum values displayed as whiskers. Mann-Whitney U-Test was performed with samples compared to WT culture: v TPx-1 KO Culture (Trx-1: p = 0.6519; 1-Cys Prx: p = 0.0124); v TPx-1 KO mosquito (Trx-1: p = 0.5777; 1-Cys Prx: p = 0.0473 ). (*) indicates that 1-Cys Prx is statistically higher expressed in TPx-1KO ookinetes. Below are representative images from IFA-epifluorescence experiments.

Trx-1 expression and distribution was comparable in culture-derived as well as in mosquito-derived TPx-1KO ookinetes (Figure 5A). Quantitative RF analysis also indicated comparable Trx-1 protein levels between TPx-1KO and WT ookinetes.

Interestingly, an accumulation of Trx-1 protein at the apical end of the protrusion was observed in early and late retorts in culture-derived as well as in mosquito-derived TPx-1KO ookinetes. These accumulations were not observed in mature ookinetes.

1-Cys Prx was not detected in early or late retort stages of culture-derived TPx-1KO ookinetes (Figure 5B). However, mature TPx-1KO ookinetes showed a strong cytosolic expression of 1-Cys Prx (median RF = 1.2), which was significantly higher than in culture-derived WT ookinetes at 24 hours. In mosquito-derived TPx-1KO ookinetes, 1-Cys Prx was already detected at significant levels in early retort stage (3 hours pIBM). Expression was maintained in the subsequent developmental ookinete stages (median RF = 2.2) (Figure 5B). Accumulation of 1-Cys Prx was also observed in the apical region of the mosquito-derived TPx-1KO ookinetes.

## Discussion

Cellular adaptation to new environmental challenges generally occurs on the transcriptional level and leads to a direct increase in functional protein [40]. The developing *Plasmodium* ookinete in the mosquito bloodmeal has to withstand an increasingly hostile environment for several hours before it reaches its motile stage. The bloodmeal contains a complex combination of cytotoxic compounds, including ROS and RNS ([5]–[7], [36], [37]. This would require the ookinete to initiate its stress response in order to maximize its chances of survival. Recent reports have shown that asexual *P. falciparum* parasites respond to oxidative challenges by upregulating various stress response genes and proteins, including *trxr*, *tpx-1* and *1-cys prx* [22], [41]. This indicates that the parasite has the capacity to respond to sudden environmental changes.

Our data confirm and expand on these observations. We have shown for the first time that antioxidant gene expression is transiently upregulated during parasite development in the mosquito bloodmeal. This upregulation occurred rapidly and peaked between 6 and 12 hours pIBM followed by a steady decline to almost initial levels. In contrast, under ookinete culture conditions we found *tpx-1* upregulation to be less strong, while an increase in *1-cys prx* transcription was not detected before 24 hours post culture setup. It is likely that the differential expression between mosquito-derived and culture-derived parasites is directly linked to the environmentally more stressful conditions in the mosquito bloodmeal. Complementing this hypothesis are recent observations showing that mosquito-derived ROS and RNS increase in the mosquito bloodmeal over time and reach a maximum at about 12 hours post bloodmeal (pBM), followed by a continuous decrease [7], [37], [42]. The transcription profiles of *trxr*, *tpx-1* as well as *1-cys prx* mirror these observations and suggest that oxidants in the bloodmeal stimulate antioxidant gene upregulation. Our results showing induction of *tpx-1* and *1-cys prx* upregulation in ookinete cultures in response to ROS challenge further support these observations.

We recognize that recent antioxidant studies on *P. falciparum* were conducted on synchronized asexual parasite cultures [22], [41], whereas our transcription data originates from mixed parasite populations under different and, in the case of the mosquito bloodmeal, varying environmental conditions. Consequently, the population dynamics differ significantly between the mosquito bloodmeal and the ookinete culture environment. For example, the majority of the parasites ingested by the mosquito will be dead or dying by 24 hours pIBM [2], while under culture conditions most parasites will survive the first 24 hours. The attrition of parasites in the bloodmeal may also contribute to the decrease in the transcription levels of *trxr*, *tpx-1* and *1-cys prx*. Yet, the expression profiles reported here were highly reproducible for each gene, indicating that most *Plasmodium* life stages respond to the bloodmeal, including developing ookinetes.

The observed upregulation of the peroxiredoxin genes in culture-derived parasites is most likely due to a slow accumulation of ROS and RNS in the culture medium. Sources of pro-oxidants include actively metabolizing cells in addition to dead as well as dying cells. Remarkably, *trx-1* did not exhibit significant modulation under either experimental condition. Yet, *trx-1* transcripts were detected at high abundance in all investigated samples (Figure S1). This may reflect the central role of *trx-1* in the redox metabolism of the malaria parasite [11], [12].

The fact that the observed transcriptional and translational upregulation of each gene occurred simultaneously makes it unlikely that these peroxiredoxins are post-translationally regulated [43], [44]. It was furthermore important to show that the observed antioxidant response in the mixed parasite population occurs in the developing ookinete since the main role of this stage is to escape the blood bolus and establish infection of the mosquito vector.

Here we show for the first time expression of Trx-1, TPx-1 and 1-Cys Prx in developing *Plasmodium* ookinetes isolated from mosquito midguts [26], [29], [35]. The increase in TPx-1 and 1-Cys Prx protein levels in mosquito-derived ookinetes corresponded very well with the observed transcriptional profile, with the distinction that, following upregulation, protein levels were maintained in all subsequent developmental stages. Trx-1, on the other hand, was present at continuous levels throughout ookinete development in mosquito-derived as well as culture-derived parasites, which was also in accordance with the observed transcriptional profile. These data strongly indicate that the expression of the thioredoxin-dependent antioxidant system is not post-transcriptionally regulated in developing ookinetes. Furthermore, ookinetes directly respond to the changing conditions in the bloodmeal by modulating antioxidant protein levels. This supports our hypothesis that the parasite possesses an environmentally regulated oxidative stress response.

The continuous and ubiquitous expression of Trx-1 observed in gametocytes, gametes and ookinetes expands on previous reports that show ubiquitous Trx-1 expression in asexual stages of *P. falciparum* (PfTrx-1) [26]. Also mass-spectroscopy based proteomics data indicate Trx-1 expression in gametocytes and mature cultured ookinetes of *P. berghei* and *P. gallinaceum*, respectively [45], [46]. Our data therefore support the proposed central role of Trx-1 in the metabolism of the malaria parasite [11], [12], [47], [48]. Since catalase and glutathione peroxidase are absent in *Plasmodium* [18] it is likely that peroxiredoxins and thus Trx-1 play a more prominent role in the antioxidant defense of the parasite [15]. Indeed, recent studies strongly indicate that PfTrx-1 interacts with multiple target proteins *in vitro* as well as *in vivo*, most notably PfTPx-1 and Pf1-Cys Prx [12], [14], [28].

Our data also show constitutive expression of TPx-1 in *P. berghei* mosquito-derived and culture-derived developing ookinetes as well as in gametocytes and exflagellating gametes. These observations expand on previous studies that show TPx-1 expression in cultured ookinetes of *P. yoelii* [35]. Mass spectroscopy- based proteomics data also indicates TPx-1 expression in cultured mosquito stages of *P. berghei* [45] and *P. gallinaceum* [46]. These data support the hypothesis of *tpx-1* as a housekeeping antioxidant gene that is primarily active against exogenous ROS and RNS [15], [23]. As such, TPx-1 expression would be under developmental rather than environmental control and thus relatively independent of exogenous conditions. However, challenging parasites with exogenous ROS leads to the upregulation of tpx-1 transcript as well as protein [22], [41]. We confirmed these findings when challenging culture-derived parasites with ROS. Our studies also show that *tpx-1* transcription and translation is elevated in mosquito-derived parasites when compared to culture-derived parasites, indicating that environmental conditions have an effect on the regulation of this gene. Taken together, these observations suggest that the parasite is continuously exposed to varying concentrations of ROS and RNS throughout its complex life cycle.

Differential regulation at the transcriptional and the translational level under different environmental conditions was most evident for 1-Cys Prx. While the enzyme was hardly detectable in developing cultured ookinetes, we found strong fluorescence signals in ookinetes isolated from mosquito bloodmeals. The timely increase in protein signal coincided with transcriptional upregulation of the *1-cys prx* gene, indicating a direct response of the parasite to the bloodmeal conditions. A lack of *1-cys prx* upregulation and protein expression was previously reported for gametocytes and cultured ookinetes of several *Plasmodium* species, including *P. berghei* [35], [45], [46]. Conversely, 1-Cys Prx is strongly expressed during asexual development of the parasite [29], [35], [49]. It was also shown that the enzyme interacts with heme *in vitro* [33]. It has therefore been hypothesized that 1-Cys Prx constitutes a bloodstage specific enzyme involved in the detoxification of ROS that originate from the parasites' food vacuole during hemoglobin (Hb) digestion [15], [33]. The absence of 1-Cys Prx in sexual and mosquito stages would then be plausible, as Hb digestion has been completed.

We demonstrate that 1-Cys Prx expression is not limited to the asexual stages and may therefore have a broader function as an antioxidant enzyme throughout the parasite life cycle. Furthermore, it can be upregulated by the parasite in response to changing environmental conditions, which is evident from the diverse transcription and protein expression profiles in mosquito-derived and culture–derived parasites. Our challenge data also suggest that *1-cys prx* upregulation may not only be dose-dependent but that a threshold exists that triggers the response in the parasite. Hence, no 1-Cys Prx expression was detected in culture-derived ookinetes as ROS build-up is comparatively slow and initially buffered by TPx-1, which shares substrate specificities with 1-Cys Prx [19], [23], [49]. In contrast, ROS concentrations rise more quickly and more strongly in the bloodmeal of the mosquito [5], [37] and thus trigger an adaptive antioxidant response in the ookinetes involving both peroxiredoxins.

This hypothesis is further supported by our studies on TPx-1KO parasites. The fact that the disruption of the *tpx-1* gene is not lethal in either *P. falciparum* [30] or in *P. berghei* [31], [32] suggested that compensatory mechanisms facilitate parasite survival. While Trx-1 expression did not vary significantly between WT and KO ookinetes, we found that 1-Cys Prx expression was significantly altered in the KO ookinetes. Specifically, enzyme expression was detected significantly earlier in mosquito-derived ookinetes. We also found higher levels of 1-Cys Prx protein in mature culture-derived KO ookinetes, which we attribute to an early upregulation between 12 and 24 hours post culture setup. We suggest that the absence of TPx-1 leads to a quicker build-up of ROS and RNS in the parasite and thus triggers an earlier upregulation of 1-Cys Prx in the KO parasite. Considering our data and the overlapping substrate specificities of the cytosolic peroxiredoxins, we hypothesize that 1-Cys Prx significantly contributes to the compensation for the loss of TPx-1 in the KO parasites. A recent report shows that the mitochondrial homolog of TPx-1, *P. berghei* thioredoxin peroxidase-2 (TPx-2), is not essential for parasite development in the mammalian host or in the mosquito vector [21], [50]. While the lack of a phenotype in the TPx-2KO parasites may be due to compensatory function of other mitochondrial redox proteins [26] it is also possible that ROS leaking from the mitochondria may be detoxified by the cytosolic system. It will therefore be highly informative to investigate the impact of TPx-2KO on the expression of other redox genes and their corresponding proteins.

The present work shows that the *Plasmodium* ookinete utilizes an inducible antioxidant response during adaptation to the challenging and changing conditions in the mosquito bloodmeal. This indicates that TPx-1 and 1-Cys Prx play overlapping roles, and that 1-Cys Prx has the capacity to compensate for the loss of TPx-1. While TPx-1 and 1-Cys Prx have similar antioxidant functions, the respective transcriptional as well as protein expression profiles indicate that these enzymes may be regulated independently. For example, TPx-1 may be inducible by lower ROS concentrations, which would explain its profile in culture-derived parasites. 1-Cys Prx upregulation on the other hand requires higher ROS concentrations; hence the late transcriptional increase in ookinete cultures. Conversely, in the mosquito bloodmeal ROS concentrations rise much more quickly and thus trigger an earlier *1-cys prx* upregulation. Alternatively, expression of each peroxiredoxin may be oxidant specific and certain compounds accumulate faster in the mosquito bloodmeal than in the culture medium, such as peroxynitrite (ONOO^−^) [37]. Upregulation of 1-Cys Prx seems to be ookinete specific, as we detected only low levels of the enzyme in gametocytes and no expression in male gametes in WT as well as in KO parasites.

Considering our data we also propose that a ROS sensing mechanism must be in place to facilitate antioxidant stress response in *Plasmodium*. Oxidant sensor proteins of the glutathione peroxidase family of proteins have recently been implicated in the oxidative stress response of the yeast *Saccharomyces cerevisiae* [51], *Drosophila melanogaster* [52] and human cells [53]. Although the *Plasmodium* genomes do not seem to contain a genuine glutathione peroxidase [11], [12], [15] a glutathione peroxidase-like thioredoxin peroxidase (TPx_Gl_) has been characterized in *P. falciparum* [24], [26]. TPx_Gl_ shares significant sequential as well as structural homologies with the putative redox sensor proteins described in yeast, fruit fly and human. Work in our lab is under way to investigate the potential signaling function of *Plasmodium* TPx_Gl_.

It has been stated that the mosquito stages of *Plasmodium*, specifically the ookinete, represent attractive targets for transmission intervention strategies [54]. It is therefore imperative to understand the mechanisms the ookinete utilizes to adapt to the adverse environment in the mosquito bloodmeal as these may hold the key to close the population bottleneck and to prevent transmission. Our results highlight the general significance of the oxidative stress response for all stages of the malaria parasite. They also emphasize that environmental conditions, especially for the “unprotected” mosquito-stages, must be taken into account when investigating the parasite-vector relationship in search for potential candidate genes.

## Materials and Methods

### Ethics statement

All experimental protocols were approved by the Institutional Animal Care and Use Committee (IACUC) of Loyola University Chicago (Protocol#148) following the National Institutes of Health guidelines for animal housing and care.

### Parasite maintenance and mosquito infections

*Plasmodium berghei* ANKA 2.34 wild type (WT) and peroxiredoxin-1 knock-out (TPx1KO) [31] parasites were maintained in Harlan ND4 mice for a maximum of four serial passages and passed through *Anopheles stephensi* mosquitoes. Mosquitoes were reared under standard conditions (26°C, 80% RH, 12 hrs light-dark cycle, 5% sucrose solution). Female mosquitoes 5–10 days post-emergence were used in all experiments. Exflagellation (2–4/20×) of parasites was tested prior to feeding to ascertain maturity. Mosquitoes were allowed to feed for 15 min on mice infected either with WT or TPx-1KO parasites (10%). Bloodfed mosquitoes were maintained at 21°C, 80% RH, 12 hrs light-dark cycle, 5% sucrose solution. Bloodfed mosquitoes were removed at experimental time points and midguts were dissected and prepared for fixation.

### Ookinete cultures and challenges

Gametocytes, exflagellating male gametes and ookinetes were obtained following established protocols [55]. Briefly, g*ametocytes* were obtained by pretreating mice with phenylhydrazine (Phz) and subsequently infecting them with WT or TPx-1KO parasites. On day two post-infection, gametocyte-rich blood was harvested and prepared for fixation. To obtain *gametes*, we incubated gametocyte-rich blood in ookinete medium for 15 min before fixation. Exflagellation rates peaked at two days post infection (>15 exflagellation/20× field). For *ookinetes*, the blood of three infected mice per experimental culture setup was pooled, diluted 1∶5 with ookinete medium (RPMI1640, pH 8.4), and incubated at 19°C for a maximum of 24 hrs. Ookinete development was followed by Giemsa-stained smears (average yield: 1×10^7^ ookinetes/ml culture) and harvested samples were directly fixed and prepared for confocal analysis. For ookinete cultures, we largely implemented protocols as described by Rodriguez et al. [55] with the following modifications: exflagellation rates of parasites from phenylhydrazine treated mice already peaked on day 2 post infection; blood of three infected mice per experimental culture setup was diluted 1∶5 with ookinete medium (RPMI1640, pH 8.4) and incubated at 19°C for a maximum of 24 hours; ookinete development was followed by Giemsa stained smears; culture samples were directly transferred into Tri-Reagent (MRC) for total RNA extraction. For the *challenges*: Increasing concentrations of paraquat (PQ) were added to ookinete cultures at 12 hours post setup; parasites were harvested following a 45 min incubation time at 19°C and total RNA was prepared for 1^st^ strand synthesis and RT-qPCR analysis.

### Quantitative real time RT-PCR and data analysis

Total RNA from bloodfed mosquito midguts and ookinete cultures was extracted using Tri-Reagent (MRC) according to manufacturer's instructions. Isolated RNA was treated with DNAse I (Ambion) and subsequently quantified using the Qubit RNA assay kit and the Qubit fluorometer (Invitrogen). RNA-samples were either immediately used for cDNA synthesis or flash frozen and stored at −80°C. cDNA was synthesized from total RNA with the High Capacity RNA-to-cDNA kit (Applied biosystems) using random hexamer primers. Sequences of target genes for primer design were acquired from Plasmodb (plasmodb.org). For the qRT-PCR, target-specific primers with T_m_≈60°C were designed to yield amplicons between 80 and 140 bp (table S1). RT-qPCR was performed on a StepOnePlus machine (Applied Biosystems) using the Fast SYBR Green Master Mix in 10 µl reaction volumes. Each sample was run in triplicates and yielded highly comparable C_t_ values (cycle threshold). No primer dimers were detected and amplicons exhibited optimal efficiencies. To test specificity, all primer pairs were tested on uninfected mouse blood, non-fed and uninfected bloodfed mosquitoes. No amplification products could be detected. Expression data was subsequently analyzed with the StepOne Software v2.2 (Applied Biosystems) and normalized against the expression of 18 s rRNA A-Type, which has been established as an internal standard for expression analysis in *Plasmodium* mosquito stages [29], [34]. For analysis of time course expression data, the ΔΔC_t_ method was used with the earliest experimental time point as a reference sample (RQ = 1). Target gene transcription levels were calculated in relation to 18 s rRNA expression by determining the ΔC_t_ value for each primer pair and subtracting the measured C_t_ value of the target from the C_t_ value of the 18 s rRNA control. ΔC_t_ values were calculated to relative transcription levels using the formula 2^−ΔCt^. The Mann-Whitney U test was used to determine if gene expression at 6, 12, and 24 hours was significantly different from the gene expression at 3 hours (reference point). The Mann-Whitney U test was conducted on each candidate gene from both mosquito-derived and from culture-derived parasites. Significance was assessed at p<0.1 due to the small sample sizes. The non-parametric Mann-Whitney U test was the appropriate statistical analytical approach to use on this dataset due to the violated assumptions of independence in the data and the small and unbalanced sample sizes in gene expression collected at 3, 6, 12, and 24 hours. Statistical analyses were performed in R [56].

### Sampling and fixation of parasite cells

*Bloodstage parasites* were isolated from tail vein blood from mice and were resuspended in RPMI 1640 pH 7.4 media with heparin pre-warmed to 37°C and allowed to adhere to 0.01% poly-L-lyseine coated glass slides at 37°C. *Gametogenesis* was induced by resuspending tail blood in RPMI 1640 pH 8.2 media with heparin pre-warmed to 19°C and allowed to adhere to 0.01% poly-L-lyseine coated glass slides at 19°C. Cells were then fixed in 3% para-formaldehyde with 0.1% glutaraldehyde at 4°C. *Cultured ookinetes* were taken at experimental time points 3, 12 and 24 hours post culture setup. RBCs were lysed with 0.17 M NH_4_Cl. Cells were then resuspended in 0.05 M Tris 0.9% NaCl pH 8.2 and allowed to adhere to 0.01% poly-L-lyseine coated glass slides. Cells were then fixed in 4% para-formaldehyde at 4°C. *Midgut ookinetes* were isolated from dissected midguts by crushing midguts with mortar and pestle in 0.05 M Tris 0.9% NaCl pH 8.2 and allowed to adhere to 0.01% poly-L-lyseine coated glass slides. Cells were then fixed in 4% para-formaldehyde at 4°C. Slides were stored at −80°C.

### Immunofluorescence staining

Slides were allowed to permeabilize and thaw in 0.05% Triton X-100 in 1× PBS. Slides were then blocked with 4% Bovine Serum Albumin. Epitopes for each protein of interest were retrieved by incubating slides in 10 mM Sodium Citrate, 0.05% Tween 20, pH 6.0 at 95°C. Autofluorescence was quenched by incubating slides in 1% Sodium Borohydride in 1× PBS. Respective antigens were detected with custom polyclonal anti-sera (1∶300) raised in rabbits (TPx-1, Trx-1, 1-Cys Prx, Open Biosystems) and labeled with anti-rabbit Alexa Fluor 488 (1∶1000) (Molecular Probes). Slides were counterstained with TO-PRO 3 (Invitrogen) and sealed with glass cover slips in ProLong Gold Antifade with DAPI mounting medium (Invitrogen).

### Confocal microscopy and quantitative analysis of relative fluorescence

Images were acquired on FluoView FV1000 Confocal Laser Scanning Microscope (Olympus). Images were taken in XY plane and compressed around the Z axis. Images in figures are representative of 50 ookinetes per time point and environment. FV10-ASW 3.0 microscopy software (Olympus) was used to analyze the samples. Quantitative fluorescence images were acquired on Zeiss Axiovert 200 m using a Axiocam MRm. Images were processed using Axiovision Rel. 4.3 software. For quantitative analysis of ookinetes, DAPI was excited by exposure at 359 nm for 20 milliseconds and AF 488 was excited by exposure at 488 nm for 350 milliseconds. Fluorescent emissions were recorded at 461 and 530 nm respectively. Images were then quantitated using Image J Software (National Institute of Health). Fluorescent Intensities for DAPI and AF488 were assessed by using the histogram function. The ratio of candidate protein fluorescence (AF 488) to DAPI fluorescence was calculated for WT culture (n = 50), TPx-1 KO culture (n = 50), WT mosquito (n = 25), and TPx-1 KO (n = 23).

### Accession numbers

PlasmoDB ID numbers for reported genes and proteins are as follows: Thioredoxin Reductase (TrxR;PBANKA_082470), Thioredoxin-1 (PBANKA_132090); Thioredoxin peroxidase-1 (TPx-1; PBANKA_130280), 1-Cysteine Peroxiredoxin (1-Cys Prx; PBANKA_122800).

## Supporting Information

Figure S1 **Relative transcript abundance of target gene transcripts in culture-derived and in mosquito-derived parasites at the 12 hours time point.** RT-qPCR data show relative quantity of target gene transcripts normalized to 18 s rRNA A-type expression [29], [34]. The delta C_t_ values were converted using (2^−ΔCt^)* 10^6^. Shown are mean values of 3 independent experiments. Error bars indicate STDEV.
(TIF)

Figure S2 **Cloning, expression and purification of** ***P. berghei*** **Trx-1 and 1-Cys Prx and specificity of the polyclonal antibodies.** **A**) Translation maps of putative Trx-1 (PBANKA_132090) and putative 1-Cys Prx (PBANKA_122800). The start codons are indicated in bold. Forward and reverse primers are underlined. The characteristic active site motifs including the peroxidatic cysteines are highlighted in yellow. **B**) Recombinant protein expression and purification. Gene specific primers were designed and PCR was performed to amplify the coding sequences using the following conditions: 40 cycles of 95°C for 30 s, 1 min at 54°C, and 45 s at 63°C. This was followed by a 5 min final extension at 63°C. The verified PCR products were ligated into pQE30 expression vectors (Quagen) and subsequently transformed into *E. coli* M15 expression cells. Recombinant protein expression was induced by adding isopropyl thio B-galactoside (IPTG) to a final concentration of 1 mM. Bacteria were harvested after a 24-hour incubation time at 37°C. Recombinant proteins were purified via a Ni-NTA column (Life technologies). Protein purity was confirmed via sodium dodecyl sulfate (SDS) polyacrylamide gel electrophoresis. Protein concentrations were assessed via Bradford Assay. Purified recombinant Trx-1 (top) 1-Cys Prx (bottom) from *E. coli*: SDS gel analysis: lane 1) protein ladder, lane 2) 10% SDS gel showing purified HIS-tagged rPb 1-Cys Prx. Lanes 3 to 5 are Western blots testing the primary antibody-containing rabbit antiserum (1/500): lane 3) protein ladder, lane 4) Western blot on purified HIS-tagged rPb 1-Cys Prx, lane 5) Western blot on *P. berghei* lysate of mixed asexual stages from mouse blood.
(TIF)

Figure S3 **Assessment of nuclear stains in ookinetes.** The fluorescence intensity of nuclear dyes DAPI and TO-PRO-3 were compared between ookinete nuclei from mixed populations of culture and mosquito (n = 15 each). DAPI (blue, left panel) was selected as the nuclear dye of choice for QF due to its lower standard error when compared to TOPRO-3 (red, right panel) (SE = 5.86 vs. 14.57).
(TIF)

Figure S4 **Protein expression of Trx-1, TPx-1 and Peroxiredoxins in gametocytes and male exflagellating gametes.** *P. berghei* gametocytes **A**) and exflagellating microgametes **B**) were differentiated by morphology. Polyclonal antisera specific for target protein Thioredoxin-1 (Trx-1), Peroxiredoxin-1 (TPx-1) and 1-Cys Peroxiredoxin (1-Cys Prx) were labeled with donkey anti-rabbit AF 488 (Molecular Probes). Cells are counterstained with TO-PRO-3. Images are merged and overlaid onto the respective DIC image. M designates merozoites also pictured and G designates gametocyte. Scale bar indicates 5 µM.
(TIFF)

Table S1 **Orthologous genes in** ***P. falciparum*** **and** ***P. berghei*** **primer sequence information.** **A**) Orthologous genes in *P. falciparum*. **B**) *P. berghei* RT-qPCR primer sequences. **C**) Protein expression primer sequences.
(DOCX)
