# Supplementary figures and images for: Expression of Cytosolic Peroxiredoxins in Plasmodium berghei Ookinetes Is Regulated by Environmental Factors in the Mosquito Bloodmeal
Source: PLoS Pathog. 2013 Jan 31;9(1):e1003136. doi: 10.1371/journal.ppat.1003136 (PMC3561267; doi:10.1371/journal.ppat.1003136)

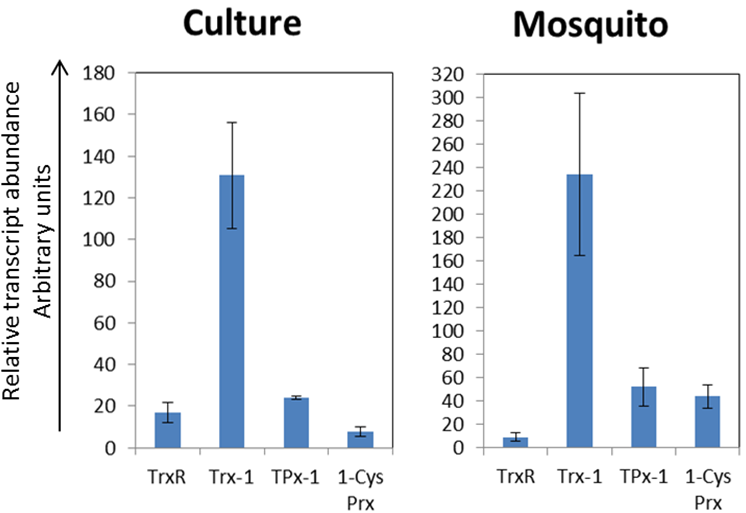

Supplement: Figure S1 — Relative transcript abundance of target gene transcripts in culture-derived and in mosquito-derived parasites at the 12 hours time point. RT-qPCR data show relative quantity of target gene transcripts normalized to 18 s rRNA A-type expression [29], [34]. The delta Ct values were converted using (2−ΔCt)* 106. Shown are mean values of 3 independent experiments. Error bars indicate STDEV. (TIF) [file ppat.1003136.s001.tif]

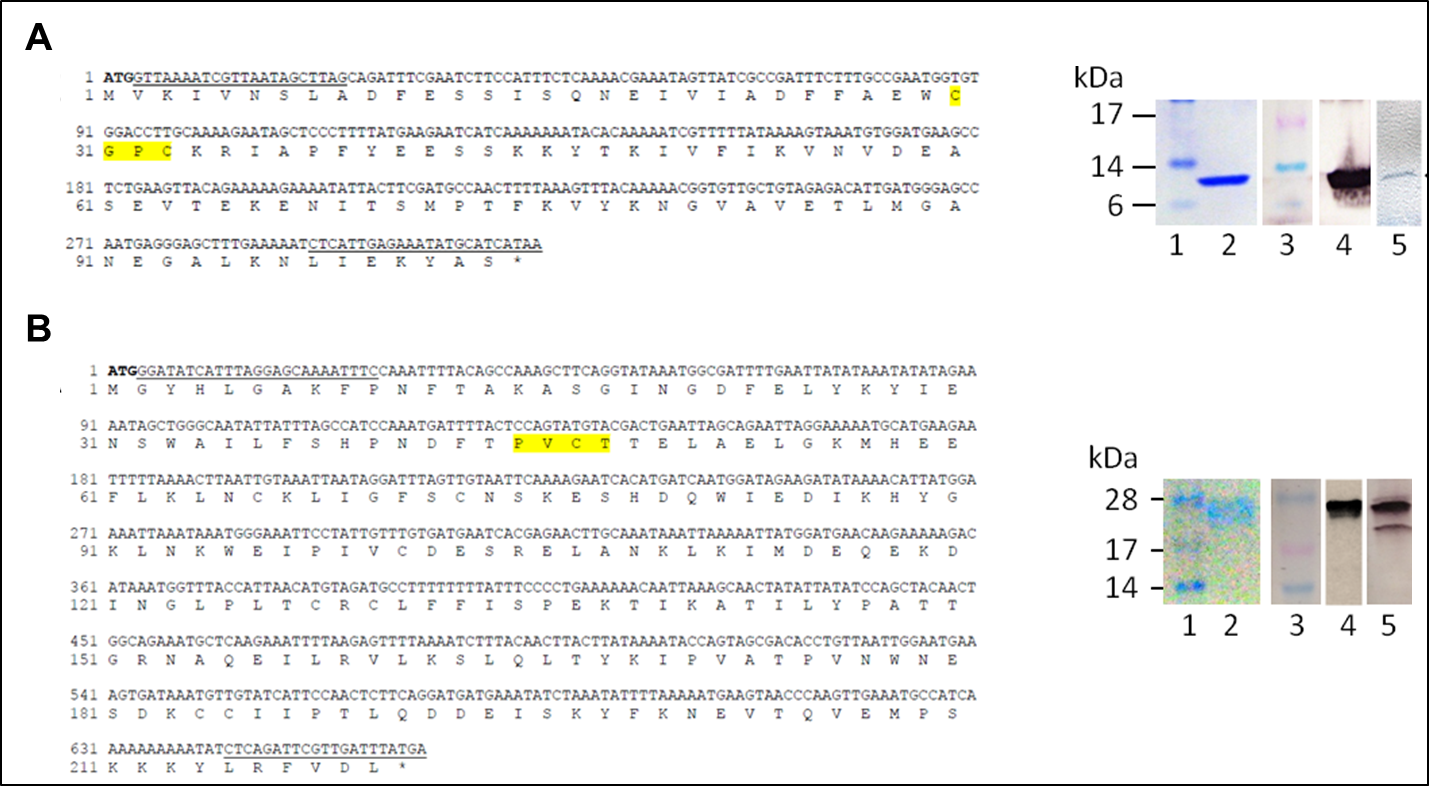

Supplement: Figure S2 — Cloning, expression and purification of P. berghei Trx-1 and 1-Cys Prx and specificity of the polyclonal antibodies. A) Translation maps of putative Trx-1 (PBANKA_132090) and putative 1-Cys Prx (PBANKA_122800). The start codons are indicated in bold. Forward and reverse primers are underlined. The characteristic active site motifs including the peroxidatic cysteines are highlighted in yellow. B) Recombinant protein expression and purification. Gene specific primers were designed and PCR was performed to amplify the coding sequences using the following conditions: 40 cycles of 95°C for 30 s, 1 min at 54°C, and 45 s at 63°C. This was followed by a 5 min final extension at 63°C. The verified PCR products were ligated into pQE30 expression vectors (Quagen) and subsequently transformed into E. coli M15 expression cells. Recombinant protein expression was induced by adding isopropyl thio B-galactoside (IPTG) to a final concentration of 1 mM. Bacteria were harvested after a 24-hour incubation time at 37°C. Recombinant proteins were purified via a Ni-NTA column (Life technologies). Protein purity was confirmed via sodium dodecyl sulfate (SDS) polyacrylamide gel electrophoresis. Protein concentrations were assessed via Bradford Assay. Purified recombinant Trx-1 (top) 1-Cys Prx (bottom) from E. coli: SDS gel analysis: lane 1) protein ladder, lane 2) 10% SDS gel showing purified HIS-tagged rPb 1-Cys Prx. Lanes 3 to 5 are Western blots testing the primary antibody-containing rabbit antiserum (1/500): lane 3) protein ladder, lane 4) Western blot on purified HIS-tagged rPb 1-Cys Prx, lane 5) Western blot on P. berghei lysate of mixed asexual stages from mouse blood. (TIF) [file ppat.1003136.s002.tif]

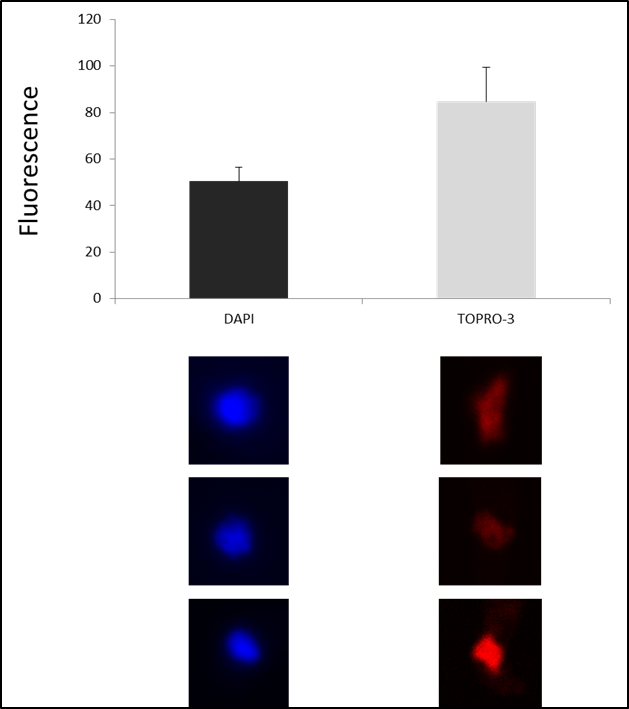

Supplement: Figure S3 — Assessment of nuclear stains in ookinetes. The fluorescence intensity of nuclear dyes DAPI and TO-PRO-3 were compared between ookinete nuclei from mixed populations of culture and mosquito (n = 15 each). DAPI (blue, left panel) was selected as the nuclear dye of choice for QF due to its lower standard error when compared to TOPRO-3 (red, right panel) (SE = 5.86 vs. 14.57). (TIF) [file ppat.1003136.s003.tif]

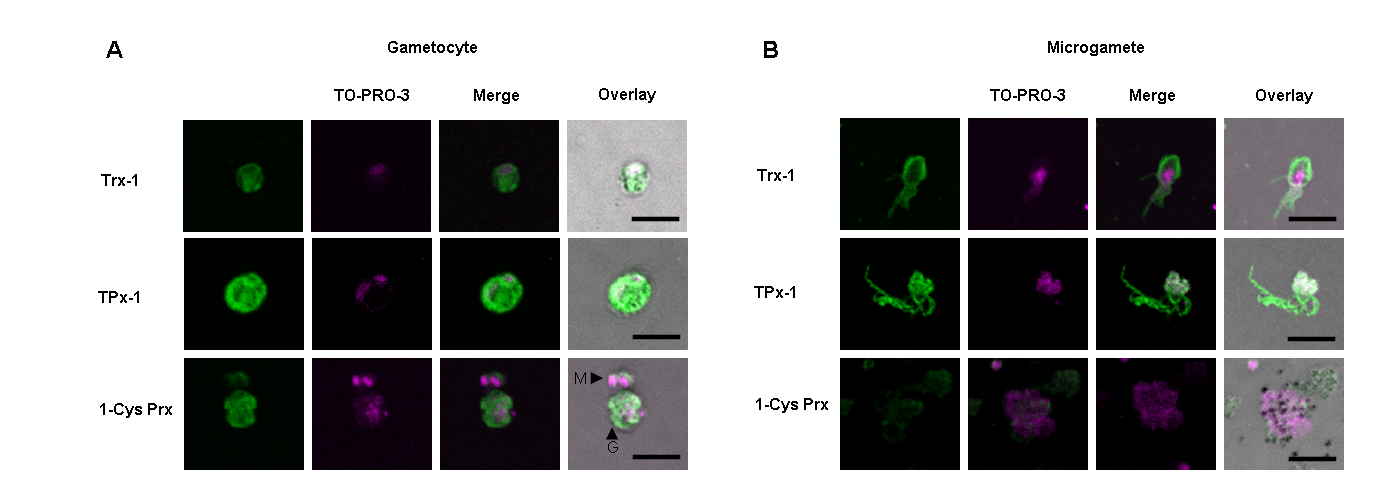

Supplement: Figure S4 — Protein expression of Trx-1, TPx-1 and Peroxiredoxins in gametocytes and male exflagellating gametes. P. berghei gametocytes A) and exflagellating microgametes B) were differentiated by morphology. Polyclonal antisera specific for target protein Thioredoxin-1 (Trx-1), Peroxiredoxin-1 (TPx-1) and 1-Cys Peroxiredoxin (1-Cys Prx) were labeled with donkey anti-rabbit AF 488 (Molecular Probes). Cells are counterstained with TO-PRO-3. Images are merged and overlaid onto the respective DIC image. M designates merozoites also pictured and G designates gametocyte. Scale bar indicates 5 µM. (TIFF) [file ppat.1003136.s004.tiff]
