# Supplementary material for: Expression of Cytosolic Peroxiredoxins in Plasmodium berghei Ookinetes Is Regulated by Environmental Factors in the Mosquito Bloodmeal
Source: PLoS Pathog. 2013 Jan 31;9(1):e1003136. doi: 10.1371/journal.ppat.1003136 (PMC3561267; doi:10.1371/journal.ppat.1003136)
Supplement: Table S1 — Orthologous genes in P. falciparum and P. berghei primer sequence information. A) Orthologous genes in P. falciparum. B) P. berghei RT-qPCR primer sequences. C) Protein expression primer sequences. (DOCX) [file ppat.1003136.s005.docx]

**Table S1 Orthologous genes in *P. falciparum* and *P. berghei* primer sequence information.**

**A** Orthologous genes in *P. falciparum* display a high degree of sequence conservation.

| **PlasmoDB** | **P.f.** | **P.f./P.b.** |
| --- | --- | --- |
| **Gene ID** | **orthologues** | **%identity** |
| PBANKA_082470 | PF3D7_0923800.1 | 79 |
| PBANKA_132090 | PF3D7_1457200 | 79 |
| PBANKA_130280 | PF3D7_1438900 | 81 |
| PBANKA_122800 | PF3D7_0802200 | 75 |

**B** *P. berghei* RT-qPCR primer sequences.

| *RT-qPCR primers* | |  |  |  |  |  |
| --- | --- | --- | --- | --- | --- | --- |
|  |  |  |  |  |  |  |
| **Putative** |  |  | **Primers** |  | **Amplicon** | |
| **Function** | **Abbr.** | **PlasmoDB ID** | **Fwd** | **Rev** | **bp** | **Tm** |
| Thioredoxin reductase | trxr | PBANKA_082470 | CCCTGGAGGTATGGCATCAG | AGTAGTCAAGGAACAAAATGGGGTAT | 102 | 74.3 |
| Thioredoxin-1 | trx-1 | PBANKA_132090 | GATGAAGCCTCTGAAGTTACAG | GGTGTTGCTGTAGAGAC ATTG | 90 | 72 |
| Peroxiredoxin-1 | tpx-1 | PBANKA_130280 | GGAAAAAAACACCATTGTCACAAG | TAACGAAAGTGTAGCATTAAGAGCG | 125 | 70.5 |
| 1-Cys peroxiredoxin | 1-cys prx | PBANKA_122800 | GGGAAATTCCTATTGTTTGTGATGA | ATGTAGATGCCTTTTTTTTATTTCCC | 123 | 71.4 |
|  |  |  |  |  |  |  |
| *Endogenous Control* |  |  |  |  |  |  |
| 18s rRNA (A-Type) | 18s rRNA | berg07_18S | GGGCTCTCAAAGGGTCTGTAATTAAAAGAAC | CGAGGCGGAGCCAAGATTC | 87 | 72.5 |

**C** Protein expression primer sequences.

| *Protein expression primers* | | **Primers** (Restriction sites in **bold**) |  |  |
| --- | --- | --- | --- | --- |
| Name | PlasmoDB ID# | **Fwd** | **Rev** |  |
| Trx-1 | PBANKA_082470 | CC**GGATCC**GTTAAAATCGTTAATAGCTTAGCAGATTTCG | CC**AAGCTT**TTATGATGCATATTTCTCAATGAGATTTTTCAAAG | |
| 1-Cys Prx | PBANKA_130280 | CC**GGATCC**GGATATCATTTAGGAGCAAAATTTCC | CC**GGTACC**TCATAAATCAACGAATCTGAG | |
